# Thermal Neutron Relative Biological Effectiveness Factors for Boron Neutron Capture Therapy from In Vitro Irradiations

**DOI:** 10.3390/cells9102144

**Published:** 2020-09-23

**Authors:** María Pedrosa-Rivera, Javier Praena, Ignacio Porras, Manuel P. Sabariego, Ulli Köster, Michael Haertlein, V. Trevor Forsyth, José C. Ramírez, Clara Jover, Daniel Jimena, Juan L. Osorio, Patricia Álvarez, Carmen Ruiz-Ruiz, María J. Ruiz-Magaña

**Affiliations:** 1 Departamento de Física Atómica, Molecular y Nuclear, Facultad de Ciencias, Universidad de Granada, 18071 Granada, Spain; mpedrosa@ugr.es (M.P.-R.); jpraena@ugr.es (J.P.); msabariego@ugr.es (M.P.S.); 2 Institut Laue-Langevin, 71 Avenue des Martyrs, CEDEX 9, 38042 Grenoble, France; koester@ill.fr (U.K.); haertlein@ill.fr (M.H.); tforsyth@ill.fr (V.T.F.); 3 Partnership for Structural Biology (PSB), CEDEX 9, 38042 Grenoble, France; 4 Faculty of Natural Sciences, Keele University, Staffordshire ST5 5BG, UK; 5 Servicio de Radiofísica y Protección Radiológica, Hospital Universitario Virgen de las Nieves, Avda. Fuerzas Armadas 2, 18014 Granada, Spain; ramirezrosjc@gmail.com (J.C.R.); clarajover@gmail.com (C.J.); danjimher@correo.ugr.es (D.J.); jl.ciberbici@gmail.com (J.L.O.); 6 Departamento de Bioquímica y Biología Molecular III e Inmunología, Facultad de Medicina, Universidad de Granada, 18016 Granada, Spain; patriar@correo.ugr.es (P.Á.); mjruizm@ugr.es (M.J.R.-M.)

**Keywords:** boron neutron capture therapy, relative biological effectiveness, thermal neutrons

## Abstract

The experimental determination of the relative biological effectiveness of thermal neutron factors is fundamental in Boron Neutron Capture Therapy. The present values have been obtained while using mixed beams that consist of both neutrons and photons of various energies. A common weighting factor has been used for both thermal and fast neutron doses, although such an approach has been questioned. At the nuclear reactor of the Institut Laue-Langevin a pure low-energy neutron beam has been used to determine thermal neutron relative biological effectiveness factors. Different cancer cell lines, which correspond to glioblastoma, melanoma, and head and neck squamous cell carcinoma, and non-tumor cell lines (lung fibroblast and embryonic kidney), have been irradiated while using an experimental arrangement designed to minimize neutron-induced secondary gamma radiation. Additionally, the cells were irradiated with photons at a medical linear accelerator, providing reference data for comparison with that from neutron irradiation. The survival and proliferation were studied after irradiation, yielding the Relative Biological Effectiveness that corresponds to the damage of thermal neutrons for the different tissue types.

## 1. Introduction

Boron Neutron Capture Therapy (BNCT) is currently undergoing a renaissance that may bring this therapy closer to hospital practice [1]. This is occurring as result of data from new accelerator sources, in combination with the promising results from previous clinical trials at research reactors [2]. A couple of this new accelerator-based sources in Japan (Kyoto Research Reactor Institute, Osaka, and Southern Tohoku General Hospital, Fukushima, Japan) have already started clinical trials [3]. In addition to this, the translation of recent research results from different disciplines to the clinical treatment may improve the therapeutic capability of this approach in the near future.

Treatment planning is one line of improvement. In a clinical study that was performed at Helsinki University Central Hospital [4], the results from two cohorts of patients receiving a differently planning tumor volume (PTV) dosing clearly showed that a small increase in the dose may lead to a much improved therapeutic outcome. The dose in BNCT, and in particular the “photon-equivalent”, “photon-isoeffective”, or “biologically weighted” [5] dose, is a key problem because the dose that the organs at risk may tolerate is a limiting factor in treatment planning and, therefore, it limits the dose delivered to PTV. Therefore, increasing the accuracy of the estimation of this dose can lead to an improvement of the clinical treatment. The biologically weighted dose is currently estimated while using fixed relative biological effectiveness (RBE) factors for the different components of the dose: thermal neutrons, fast (including epithermal) neutrons, boron, and gamma [6], although, recently, more accurate formalisms, such as the photon iso-effective dose [7] and intermediate models, have been proposed [8]. In all of these models, either the RBE factors or the alpha and beta radiobiological coefficients are required for both the BNCT dose components and for reference photon irradiation. Therefore, it is desirable to independently determine the experimental response to neutrons in the three energetic groups that were considered for BNCT: thermal (*E_n_* < 0.5 eV), epithermal (0.5 eV ≤ *E_n_* ≤ 10 keV), and fast (*E_n_* > 10 keV). 

A source of information of the response of cells to neutrons are in-vitro irradiation experiments, where different cell cultures are irradiated in a neutron beam at different doses and the biological end-point considered (the “survival”) is the fraction of cells that has the ability to form clones a few days after the irradiation (clonogenic assay). From the comparison of these survival curves from neutron irradiation with those that were obtained from a reference photon irradiation, the relative biological effectiveness of neutrons as compared to photons can be obtained. Some of these experiments can be found in Refs. [9,10,11,12,13]. However, in the previous measurements, the neutron irradiation was performed with a mixed beam that contained fast and thermal neutrons as well as gamma rays. In that case, and for estimating the pure effect of neutrons, the subtraction of the gamma component may lead to significant uncertainties if its contribution is large. Additionally, due to the mixing of fast and thermal neutrons in the previous experiments, a common RBE factor for both has been adopted, whilst there has been some evidence against this assumption [14]. A spectrum-dependence of the neutron effect would mean that the RBE of the fast neutron dose could depend on the particular facility being used, and it might be very different at reactor or accelerator-based sources. On the other hand, the RBE of thermal neutrons does not depend on the energy spectrum, because the biological effect is mostly due to the high-energy products of the ^14^N(n,p)^14^C reaction (625.87 keV). Thus, in practice, a low energy neutron spectrum does not affect the energy of these products. Therefore, the RBE of thermal neutrons is a key factor in BNCT, because it does not depend on the facility. 

The aim of this work was to measure the biological response of different cell lines (cancer and normal tissues) under a pure low energy neutron beam, with almost no contamination of fast neutrons and gamma radiation, and then compare it with the response of the same cell lines under a reference photon irradiation from a hospital LINAC. Dedicated setups were designed for both kinds of irradiations. Two different biological end points have been studied for the determination of the neutron response, but only the common one (clonogenic ability) has been chosen for the thermal neutron RBE calculations.

## 2. Materials and Methods

### 2.1. Neutron Irradiations

**Figure 1:**
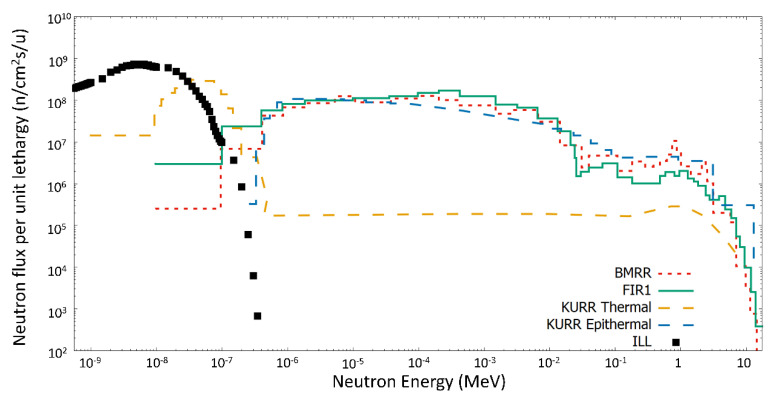
Simulated neutron spectrum at the end of the collimation system of the PF1B line at the Institut Laue-Langevin (ILL) (squares) when compared with neutron Boron Neutron Capture Therapy (BNCT) sources [17,18], such as the epithermal beam of Brookhaven Medical Research Reactor (BMRR), epithermal and thermal beams of Kyoto University Research Reactor Institute (KURR) and the epithermal beam in Finland Reactor 1 (FIR1). Data are expressed in neutron flux per unit of lethargy.

Neutron irradiations were carried out at the PF1B cold neutron beam line at the Institut Laue-Langevin (ILL) [15]. The arrangement that was developed for neutron irradiation of culture cells has been described elsewhere [16]. This cold beam (lower energy than thermal) is equivalent to a thermal neutron beam, because the energy that is delivered in the neutron interactions does not depend on its kinetic energy and, as a result of the 1/v behavior (where v is the neutron velocity) of neutron capture in the cold/thermal energy range, the thermal equivalent flux can be used to characterize the beam [15]. Hence, in the following we discuss “thermal dose” even if the neutron spectrum used was actually “cold”. On PF1B, the epithermal neutron and gamma contributions are negligible as a result of the bent guide. The thermal equivalent neutron flux at the sample position, as measured by gold foil irradiation, was 1.75 × 10^9^ n_thermal_/(cm^2^s). Figure 1 shows how this low energy spectrum, without any fast neutron contribution, is ideal for studies of the thermal factor, in contrast to the epithermal neutron beams used in BNCT.

**Figure 2:**
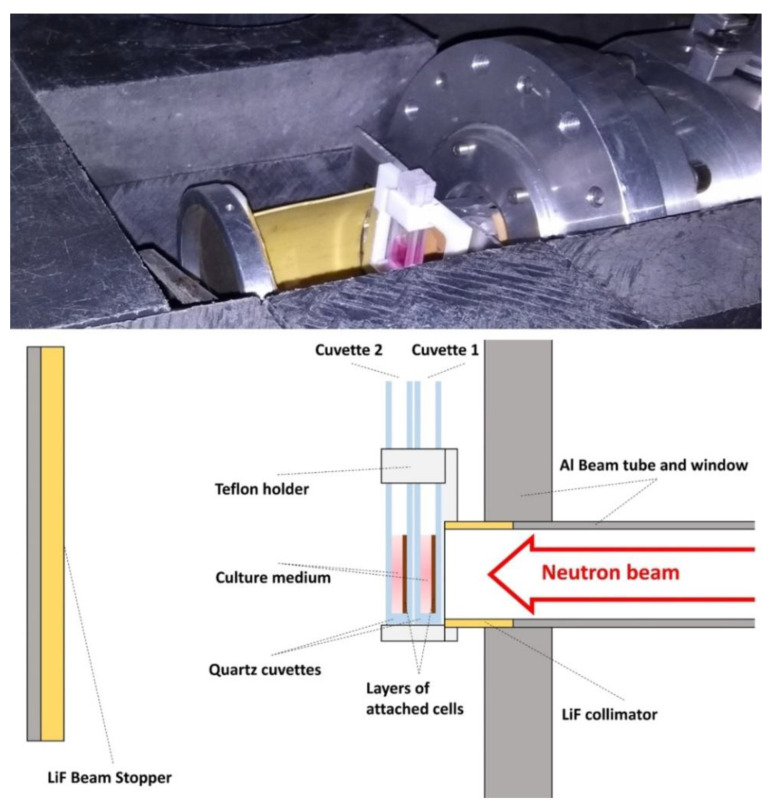
Picture and schematic cut view of the experimental arrangement installed on the PF1B instrument at the institute Laue-Langevin (ILL). Two cuvettes containing cells were irradiated at the same time. All of the cells are irradiated homogeneously and the second cuvette receives a smaller dose than the first one. LiF is used as the first layer of collimators and as a beam stop, capturing neutrons without the generation of secondary gamma radiation.

The experimental arrangement that is shown in Figure 2 with two consecutive quartz cuvettes was designed for their simultaneous irradiation with the second one receiving lower dose than the first one. In each cuvette, the cells were attached to the surface perpendicular to the neutron beam. The gamma dose is due to the neutron capture on the elements of the quartz and the cell culture medium and it stays lower than the neutron dose, which makes this instrument a very appropriate place to study the effect of low-energy neutrons. The cells were irradiated homogenously during times ranging from 15 to 75 min. and at room temperature (23–25 °C). 

### 2.2. Photon Irradiations at Medical Linear Accelerator

**Figure 3:**
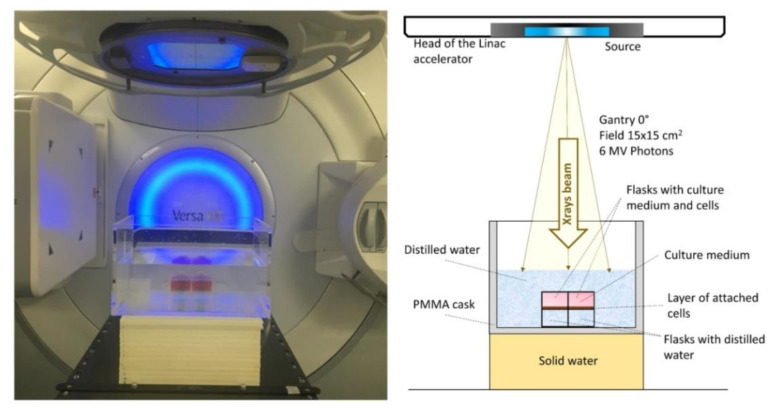
Experimental arrangements for cell irradiations with photons used at the medical linear accelerator in Granada. Two flasks with a layer of cells are irradiated at the same time with the same dose.

Photon irradiations were carried out at the medical linear accelerator (LINAC) of Virgen de las Nieves Hospital (HVN) in Granada. The Elekta Versa HD™ accelerator delivered a flattened 6 MV high energy photon beam [19]. The irradiation zone was adapted in order to carry out in vitro irradiations. The electronic equilibrium was secured by immersing the flasks in distilled water and placing under these 14 cm of solid water (see Figure 3). Two flasks were simultaneously irradiated at room temperature (23–25 °C), both within the field of the beam, and then receiving the same dose. Irradiations of 0.5–6 Gy were performed at 1 Gy/min. dose rate. 

### 2.3. Dose Estimation

Measurements of neutron flux were performed using gold foil activation data. Neutron doses at the cells were estimated with the MCNPX simulation code for the transport of the neutrons and the generated photons [20]. The geometry of the experimental arrangement was accurately simulated, calculating the dose by means of the kerma factor that depends on the nitrogen content of each cell line. Corrections due to charged particle disequilibrium effects, which are important, as shown by Bortolussi et al. [21], were included in the dose estimations. More details can be found in Ref. [16].

Photon doses at HVN medical LINAC were known from a computed tomography scan image and the clinically used Pinnacle treatment planning system (Philips, Amsterdam, The Netherlands) [22], which uses a collapsed cone convolution superposition algorithm.

### 2.4. Nitrogen Content Analysis

At thermal energies, the neutron capture on nitrogen dominates the local dose deposition among all possible reactions in the tissue. Therefore, the nitrogen content of the cells is essential for the dose estimation. CHNS elemental analysis was performed with a THERMO SCIENTIFIC Flash 2000 (Thermo Fisher, Waltham, MA, USA) analyzer. CHNS elemental analyzers provide a means for the rapid determination of carbon, hydrogen, nitrogen, and sulphur in organic matrices and other types of materials. It is based on the dynamic combustion of a sample. The resultant gases are separated and detected by a thermal conductivity detector (TCD). This technique can determine the quantity of carbon, nitrogen, hydrogen, and sulphur with an error less than 3%. 

The nitrogen content of cells was measured by this method at the Centro de Instrumentación Científica (CIC) from the University of Granada. To this end, a dry pellet of 10 × 10^6^ cells was prepared and 2 μg of the pellet were used for the CHNS elemental analysis.

### 2.5. Cells and Cell Culture

The six irradiated cell lines were of human origin, four from cancer tissue and two from healthy tissue. The A375 cells are from malignant melanoma; Cal33 and SQ20 from head and neck squamous cell carcinoma; U87 from glioblastoma; HEK293 are from embryonic kidney; and, MRC5 are fibroblast from fetal lung tissue. The cell lines were kindly provided from Institute of Advanced Biosciences, Grenoble, except for HEK293, which was commercially acquired (Thermo Fisher). The selected cancer cell lines correspond to the type of tumors that have been typically treated with BNCT. All of them are adherent cells, which is also a requirement in our set-up. 

The cells were cultured in DMEM medium (HyClone, Logan, UT, USA) that contained 10% fetal bovine serum (FBS; Gibco, Carlsbad, CA, USA), 1 μM L-glutamine (Gibco), 100 IU/mL penicillin, and 100 IU/mL streptomycin (Sigma-Aldrich, St. Louis, MO, USA) (complete medium) at 37 °C in a humidified CO_2_ 95% air incubator. The cells were sub-cultured at a ratio of 1:3 and they were grown to 90% confluence. The medium was replaced every 2–3 days. 

Twenty-four hours before irradiations, cells were cultured in complete medium at 70% confluence in either quartz cuvettes (between 1.5 × 10^5^ and 2 × 10^5^ cells in 200 μL culture medium) for neutron irradiation or T25 flasks (between 0.7 × 10^6^ and 1.4 × 10^6^ cells) for photon irradiation. The medium was replaced with fresh complete medium before irradiating the cells.

### 2.6. Clonogenic Assays

The irradiated cells were detached with 1% trypsin-EDTA (Sigma-Aldrich) and then prepared for clonogenic assays. Cells were seeded in triplicate on six-well plates at appropriate numbers (between 2 × 10^2^ and 8 × 10^3^ cells/well), based on the irradiation dose and the growing characteristics of the cell line, to form colonies of more than 50 cells in around two weeks. Every four days, the medium was exchanged with fresh complete medium. Colonies were fixed with 90% ethanol, stained with crystal violet, and then counted using the open access automatic counter program by Nghia Ho [23].

### 2.7. Proliferation Assays

After irradiation, the cells were cultured in triplicate in 96-well plates at a density of 1 × 10^3^ cells/well and incubated for four days. Proliferative ability was determined by a 5-bromo-2-deoxyuridine (BrdU) ELISA kit (Roche, Mannheim, Germany), following the manufacturer’s instruction.

## 3. Results

### 3.1. Dosimetry

For ILL neutron irradiation studies, with nitrogen concentration in the cell lines ranging from 1.0 to 2.3%, the simulated neutron thermal doses rates varied from 0.005 to 0.026 Gy/min., depending on the tissue type. The elemental analyzer method is validated, as the nitrogen content of HEK293 cell line measured is in good agreement with the reference in ICRU report 46 for fetus kidney [24]. Table 1 indicates the nitrogen content for the remaining cell lines. Similar quantities of nitrogen, around 2%, are found in most of the cell lines, except for the MRC5 healthy cell line, where the amount of measured nitrogen is lower. It should be noted that the thermal dose is nearly entirely (≈96% contribution) due to neutron captures on nitrogen [25]. Hence, a variation in nitrogen concentration leads in first order to a proportional change of thermal dose. The assumed nitrogen content and resulting thermal dose are both shown in Table 1. 

Gamma doses, mainly from neutron interaction with the experimental system, are tissue-independent and, therefore, are the same for all cell lines. In most of the cases, the thermal dose component remained higher than the gamma dose component (see Table 1). The main objective of the irradiations, which was to have a dose mostly due to low-energy neutrons, was achieved. 

The biological effect of the secondary gamma radiation would be expected to be less than in higher dose rate LINAC irradiations due to the low gamma dose rate. Indeed, this could be expected in long irradiation, because of repair effects. Nevertheless, the RBE of the gamma dose is set equal to 1 due to the lack of data for the studied cell lines at the present dose rate. 

Additionally, conclusive data for the possible synergies between the high-LET and low-LET radiations were not found. Thus, a cross term for describing this effect in the survival curves was not considered. 

### 3.2. Results for Neutron Irradiation

**Figure 4:**
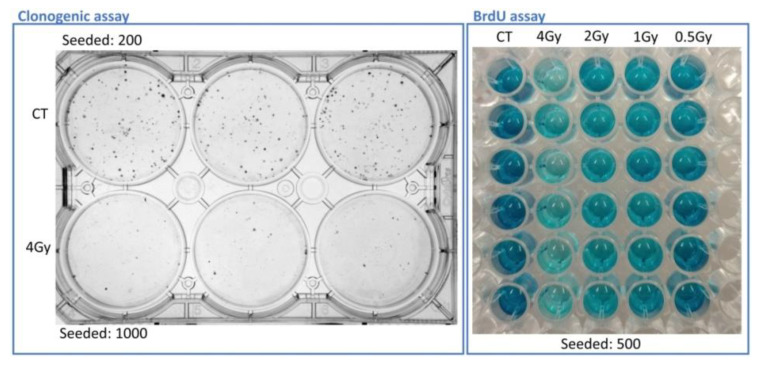
A375 plates for both clonogenic and proliferative assays. Left, plate for clonogenic assay with the control sample (CT) and the sample irradiated with a neutron dose of 4 Gy at day seven after irradiation. Right, BrdU cell proliferation assay of control and irradiated cells at day four after irradiation. The samples were analyzed in triplicate for each data point.

Two end-points were studied for the neutron irradiations: survival, by means of clonogenic assay, and proliferation, by using BrdU incorporation assay, around two weeks and four days, respectively, after the irradiation. Figure 4 displays plates for the visual appearance of these assays with A375 cells.

**Figure 5:**
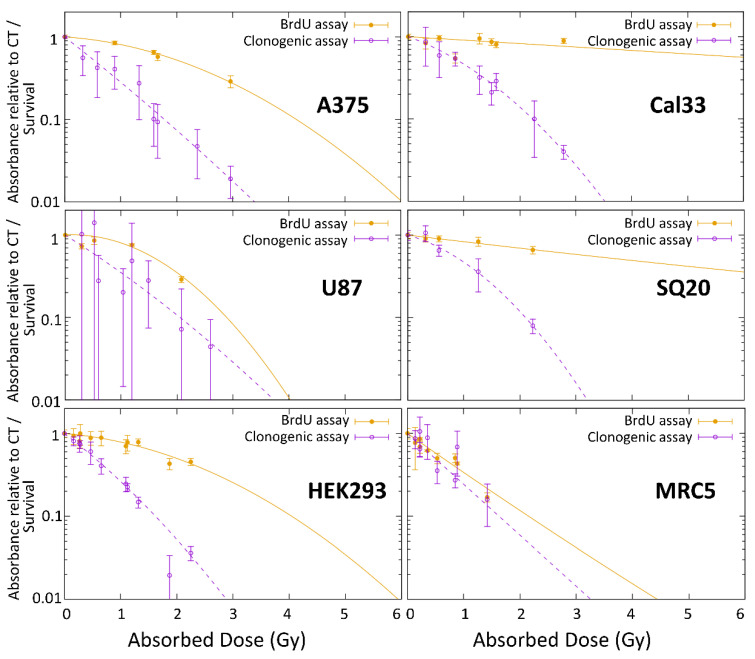
Proliferation and survival data for the different cell lines in response to neutron irradiation. Proliferation (expressed as absorbance relative to control (CT), as determined by BrdU assay), and survival (based on clonogenic assays) are represented for each cell line as a function of the total absorbed dose after neutron irradiation at ILL. Data were obtained from between two and four individual experiments with three replicate dishes plated per point per experiment.

Both end-points showed different results when data were fitted following a linear quadratic equation, as shown in Figure 5. The biggest difference between the survival and proliferation curves was observed in the head and neck cancer cell lines. 

Because a correlation between the two different end-points could not be established, probably because of the time after irradiation at which they are measured, the comparison with photon irradiation, and the calculations of RBE factors are limited to the most common end-point used, which is the long-term survival by clonogenic assay.

### 3.3. Cell Survival

Survival data after irradiation, *S*, as studied by clonogenic assays, were fitted using the linear quadratic formula [26]:

(1) $$-\ln S=\alpha D+\beta D^{2}$$

where *D* is the total absorbed dose and *α* and *β* are the parameters that describe the behavior of the survival. These parameters are constants that depend on the tissue/cell line and end-point. 

For irradiations at the medical LINAC, the total dose will correspond to that for the photons only, *D_γ_*, while the survival following irradiations at ILL is due to a dose combination of low-energy neutrons and photons, *D_ILL_*. Thus, the total absorbed dose at ILL beam corresponds to the sum of neutron and gamma dose, *D_ILL_* = *D_n_* + *D_γ_*. 

From the two fitted survival data, *S_ILL_* and *S_γ_*, the survival that corresponds to the neutron effect alone, *S_n_*, can be calculated as:

(2) $$S_{n}=\frac{e^{-\left(\alpha_{ILL}D_{ILL}-\beta_{ILL}D_{ILL}^{2}\right)}}{e^{-\left(\alpha_{\gamma }D_{\gamma }-\beta_{\gamma }D_{\gamma }^{2}\right)}}$$

This survival describes the effect if the dose is due to low-energy neutrons alone, *D_n_*. The data can be fitted to a quadratic function, but the parameter *β* is assumed to be zero given the characteristics of high-LET radiation and the better results found with a linear fitting. 

**Figure 6:**
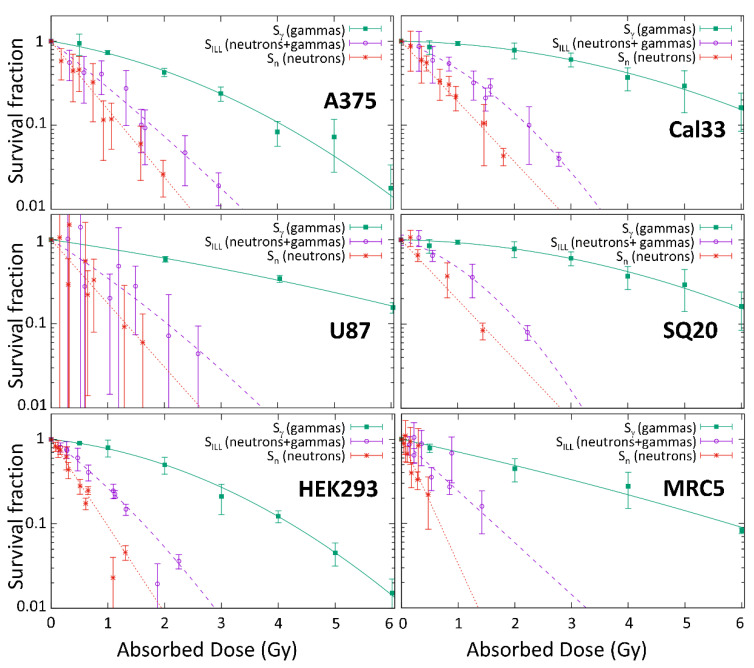
Clonogenic survival of six cell lines as a function of the absorbed dose (Gy) following irradiation with photons at the medical linear accelerator, *S_γ_*, with neutrons at the ILL beam, *S_ILL_*_,_ and, derived, ILL neutrons alone, *S_n_*. *S_γ_* for U87 from [27] and *S_γ_* for SQ20 from the one obtained for Cal33. The data were obtained from between two and seven individual experiments after photon irradiation and neutron irradiation. Cells were seeded in triplicate for each data point in each experiment.

Figure 6 shows the survival after neutrons irradiations at ILL (*S_ILL_*) and the survival after photon irradiations at HVN medical LINAC (*S_γ_*). As a result of the neutron-induced gamma production in the experimental arrangement, he survival (*S_n_*) that was associated with the neutron dose alone was obtained by the deduction of the gamma effect, as indicated in Equation (2). Table 2 shows the alpha and beta parameters for each one.

The U87 cell line was not photon irradiated and the results from Bayart et al. [27], obtained with a Varian NDI 226 X-ray tube of 200 kVp (kilovolt peak) at a dose rate of 1.2 Gy/min, were used. Additionally, the SQ20 cell line was not irradiated at the medical LINAC; data from Cal33, also squamous cell carcinoma, were used for reference. 

### 3.4. Relative Biological Effectiveness

The RBE factors corresponding to thermal neutrons mainly arise from the neutron induced proton emission from nitrogen, ^14^N(n,p)^14^C, where the emitted charged particles (protons and ^14^C) damage the tissue, as mentioned previously. The RBE of thermal neutrons are calculated from a reference irradiation dose, *D*_γ,*ref*_, and the dose corresponding to only neutrons, *D_n_*, as described by *α_n_* and *β_n_*:

(3) $$RBE_{t}\;\mathrm{or}\;w_{t}=\frac{D_{\gamma ,ref}}{D_{n}}$$

The irradiations at the medical LINAC serve as reference irradiation, as described with the *α_γ_* and *β_γ_* previously calculated. For different survival, the corresponding doses calculated with parameters in Table 2 lead to the thermal RBE factors shown in Table 3. Data that are outside the measured range are estimated by extrapolating the curves with the fitting parameters. It is evident that the uncertainty propagation of the cell survival after neutron and photon irradiation leads to huge uncertainties for the deduced RBE values for the cell line MRC5 and large uncertainties for the U87 cell line. Additional experiments with photons and with neutrons are foreseen in order to reduce this uncertainty and provide meaningful RBE values.

A new formalism for biological dose estimation has been proposed [7]. It avoids the use of variable RBE factors, and it can be considered as a simplification of the photon iso-effective dose model [6]. This model makes use of constant weighting factors, as shown in Table 4, which do not depend on the survival (or dose), defined as:

(4) $$W_{t}=\frac{\alpha_{n}}{\alpha_{\gamma }}$$

Again, the high errors on the fitting parameters of the irradiations in some of the cell lines lead to high uncertainties, mainly due to the error of the photon response, which can be reduced with more irradiation experiments. For example, in the case of Cal33 and SQ20, the error is due to the low effect that was observed after LINAC irradiations, where the fitting parameters have an error that is comparable to the value. More irradiations at LINAC may improve this result.

## 4. Discussion

Data showing the biological response of low-energy neutron irradiation show marked variability for the six cell lines studied. Therefore, it is crucial to characterize and understand the weighting factors for specific tissues and avoid having to assume such values. 

The proliferation and survival curves obtained following neutron irradiation seem to be different in most cell lines. Although this fact could suggest contradictory results depending on the end-point selected, the proliferative ability of the viable cells was assessed four days after irradiation while the clonogenic assay estimated the long-term cell survival. Moreover, proliferative and clonogenic potential do not have to necessarily correlate, as different factors may influence attachment, proliferation, and growth, such as cell density and cell-cell contact. Overall, our results suggest that it should be interesting to analyze different parameters, as they could provide more information about the possible behavior and response of cells to irradiation in vivo.

We have also shown how differences in the nitrogen content between different cell lines have a strong impact on the dose received, emphasizing the importance of the accurate estimation of the elemental composition of each tissue under the neutron field for a better BNCT treatment planning. As we have demonstrated by determining the nitrogen content of HEK293 and comparing with the literature, the CHNS elemental analyzer seems to provide a simple and useful method for measuring it.

The healthy tissue cell line MRC5 has the lowest amount of nitrogen, but high RBE values after low-neutron irradiation, which indicates an intense radio-sensibility, since less nitrogen captures took place, but a low survival was observed. 

The survival data suggest that Cal33 and SQ20, which are representative of a similar cancer type, behave in a similar way and, therefore, have similar RBE values. They both showed a strong effect after neutron irradiation (as compared to photons) and the data were markedly different from the other cell lines with similar nitrogen content, such as A375 or U87. 

The results of the A375 study suggest that this cell line appears to be more radio-resistant to neutrons than the others. The numerical values from previous work [15] are updated, accounting for the specific nitrogen content of this cell line.

The results from the U87 cell line can be compared with a similar in vitro irradiation of rat gliosarcoma cells at BMRR [11], where the RBE at 1% survival is 3.8. The value is lower than the 4.7 that was found for the U87 cells. This difference can be due to the different tissue or the different energy spectra. The neutron RBE values of a typically non-tumor tissue irradiated cell line, V79 hamster cells, vary from 1.9 to 14.5 [9,10,13], with most of them obtained from mixed epithermal beam. In the case of the non-tumor cell lines in our experiments, 3.2 and 7.6 are measured at 1% of survival for HEK293 and MRC5, respectively. In general, values found here are bigger than previous RBE values obtained in the different beams. This could suggest that the thermal RBE is bigger than when it is assumed as the same as the epithermal RBE or it could also suggest some synergies between low-LET and high-LET particles that would require further research. Values for the constant weighting factors iso-effective dose formalism, *W_t_*, [8], are much higher than the ones that correspond to the survival-dependent common formalism in Table 3. This difference is expected, since, by definition, they are the maximum values of the weighting factors in the low-dose limit. It is important to point out that this does not mean a higher biological dose, because of the different use of the weighting factor in this iso-effective formalism. 

The accuracy of the photon irradiation data is important when used as a reference radiation in the comparison with neutrons for the RBE estimation. A common type of reference photon radiation, at a specific dose rate, would be desirable. Irradiations at the medical linear accelerator at dose rates of few Gy/min. are a good option in acquiring data for the reference photon dose, providing a comparison with a standard irradiation field for which there is a lot of clinical experience. Because the U87 and SQ20 cells were not available for photon irradiations, the reference irradiation used is not from the same series of irradiation at the LINAC, like the other cell lines. This implies that these RBE results may not be directly comparable with the ones from A375, Cal33, MRC5, and HEK293 until the irradiation in the same type of photon beam is possible. However, the main results from this work are the neutron response and the radiobiological parameters $\alpha_{n}$. In the case of future refined values of the photon coefficients being found, the RBE values can be easily recalculated just by inserting the new values in Equations (2) and (3). The dose rate effect and possible synergies between high and low LET radiations have not been considered in the present work. New measurement campaigns to study these interesting effects have already been foreseen.

A very important contribution to the especially large uncertainties for the MRC5 cell line is due to the uncertainty of the β value, which makes this value compatible to zero, as it can be seen in Table 2. Although this value is obtained from a more limited set of experiments than the other cell lines, it seems that this cell line is not very representative as a model of the biological response of normal tissues, which are characterized with low α/β ratios for photon irradiation.

It has been shown how with an appropriate experimental arrangement and a clean cold neutron beam, such as the one at ILL, the radiobiological coefficients *α*_n_ for thermal neutrons can be obtained with higher precision than has been described previously. Here, they have been obtained with an uncertainty of less than 15% for all cancer and normal cell line.

The obtained data provide very interesting information on the cell types that may be more radio-sensitive to neutron irradiation, and the difference between the tissues. This could be of relevance for BNCT treatment planning when healthy tissues with different neutron sensitivity need to be considered. It would be interesting to study other non-tumor cells of different origins. 

The importance of the RBE data that were provided by a pure thermal neutron beam has been pointed out. It is stressed that thermal RBE is a universal value that does not depend on the neutron beam and neutron facility. In order to provide very accurate data, the determination of the fast and epithermal neutron RBE in each facility is extremely recommended due to the different characteristic in their spectrum. Further research work of this type on other cell lines and in vivo studies are desirable for improving the understanding of the biological response to neutrons and for the improvement for BNCT treatment planning.

## Figures and Tables

**Table 1:** Nitrogen content and dose components of the cell lines irradiated using the PF1B neutron beam instrument at the Institut Laue-Langevin (ILL). Campaign of June 2018.

Cell Line	Nitrogen Content (%)	Thermal Dose, *D_n_* (Gy/min)		Gamma Dose, *D_γ_* (Gy/min)	
		Cuvette 1	Cuvette 2	Cuvette 1	Cuvette 2
A375	2.2	0.026	0.012	0.013	0.010
Cal33	2.3	0.024	0.011	0.013	0.010
U87	2.0	0.021	0.010	0.013	0.010
SQ20	2.3	0.024	0.011	0.013	0.010
HEK293	1.7	0.018	0.008	0.013	0.010
MRC5	1.0	0.011	0.005	0.013	0.010

**Table 2:** Alpha and beta values (±standard error) for medical linear accelerator irradiations and for the different dose components at ILL.

Cell Line	ILL, Total		Medical LINAC, Photons		ILL, Pure Neutrons
	α_ILL_ (Gy^−1^)	β_ILL_ (Gy^−2^)	α_γ_ (Gy^−1^)	β_γ_ (Gy^−1^)	α_n_ (Gy^−1^)
A375	1.22 ± 0.16	0.04 ± 0.06	0.25 ± 0.03	0.075 ± 0.012	1.86 ± 0.06
Cal33	0.56 ± 0.09	0.21 ± 0.04	0.03 ± 0.02	0.047 ± 0.007	1.65 ± 0.05
U87	0.98 ± 0.43	0.07 ± 0.21	0.23 ± 0.02	0.012 ± 0.005	1.74 ± 0.19
SQ20	0.44 ± 0.27	0.31 ± 0.13	0.03 ± 0.02	0.047 ± 0.007	1.63 ± 0.13
HEK293	1.18 ± 0.10	0.14 ± 0.06	0.16 ± 0.01	0.091 ± 0.002	2.37 ± 0.09
MRC5	1.41 ± 0.50	0.00 ± 0.50	0.34 ± 0.08	0.011 ± 0.015	3.40 ± 0.45

**Table 3:** Thermal neutron relative biological effectiveness (RBE) values (*w_t_* factor) for the different cell lines irradiated at ILL as a function of the cell survival fraction.

Survival	A375	Cal33	U87	SQ20	HEK293	MRC5
50%	4.8 ± 0.7	8.4 ± 1.3	6.7 ± 2.6	8.4 ± 1.7	6.8 ± 0.4	9.5 ± 9.8
37%	4.3 ± 0.7	7.1 ± 1.2	6.4 ± 2.7	7.1 ± 1.5	6.0 ± 0.4	9.3 ± 11.0
10%	3.3 ± 0.6	4.8 ± 0.8	5.5 ± 5.6	4.8 ± 1.1	4.3 ± 0.3	8.5 ± 11.8
1%	2.6 ± 0.5	3.4 ± 0.6	4.6 ± 2.3	3.4 ± 0.8	3.2 ± 0.2	7.6 ± 11.0

**Table 4:** Constant weighting factors, *W_t_*, of the simplified iso-effective formalism [7].

Weighting Factor	A375	Cal33	U87	SQ20	HEK293	MRC5
***W_t_***	7.3 ± 1.2	57 ± 51	7.7 ± 1.6	57 ± 51	14.5 ± 1.2	10 ± 4

## References

[1] Barth R.F., Vicente M.G.H., Harling O.K., Kiger W., Riley K.J., Binns P.J., Wagner F.M., Suzuki M., Aihara T., Kato I. (2012). Current status of boron neutron capture therapy of high grade gliomas and recurrent head and neck cancer. Radiat. Oncol..

[2] Kreiner A., Bergueiro J., Cartelli D., Baldo M., Castell W., Asoia J.G., Padulo J., Sandín J.C.S., Igarzabal M., Erhardt J. (2016). Present status of accelerator-based BNCT. Pract. Oncol. Radiother..

[3] Sakurai Y. Progress in Reactor and Accelerator Based BNCT at Kyoto University Research Reactor Institute. Proceedings of the 26th International Nuclear Physics Conference.

[4] Joensuu H., Kankaanranta L., Seppälä T., Auterinen I., Kallio M., Kulvik M., Serén T. (2003). Boron neutron capture therapy of brain tumors: Clinical trials at the Finnish facility using boronophenylalanine. J. Neuro-Oncol..

[5] Sato T., Masunaga S.I., Kumada H., Hamada N. (2018). Microdosimetric Modeling of Biological Effectiveness for Boron Neutron Capture Therapy Considering Intra- and Intercellular Heterogeneity in (10)B Distribution. Sci. Rep..

[6] International Atomic Energy Agency (2001). Current Status of Neutron Capture Therapy.

[7] González S.J., Santa Cruz G.A. (2012). The photon-iso-effective dose in boron neutron capture therapy. Radiat. Res..

[8] Pedrosa-Rivera M., Praena J., Porras I., Ruiz-Magaña M.J., Ruiz-Ruiz C. (2020). A simple approximation for the evaluation of the photon iso-effective dose in Boron Neutron Capture Therapy based on dose-independent weighting factors. Appl. Radiat. Isot..

[9] Hall E.J., Novak J.K., Kellerer A.M., Rossi H.H., Marino S., Goodman L.J. (1975). RBE as a function of neutron energy: I. Experimental observations. Radiat. Res..

[10] Gabel D., Fairchild R.G., Larsson B., Börner H.G. (1984). The relative biological effectiveness in V79 Chinese hamster cells of the neutron capture reactions in boron and nitrogen. Radiat. Res..

[11] Coderre J.A., Makar M.S., Micca P.L., Nawrocky M.M., Liu H.B., Joel D.D., Slatkin D.N., Amols H.I. (1993). Derivations of relative biological effectiveness for the high-LET radiations produced during boron neutron capture irradiations of the 9L rat gliosarcoma in vitro and in vivo. Int. J. Radiat. Oncol. Biol. Phys..

[12] Gavin P.R., Kraft S.L., Huiskamp R., Coderre J.A. (1997). A review: CNS effects and normal tissue tolerance in dogs. J. Neuro. Oncol..

[13] Mason A.J., Giusti V., Green S., af Rosenschöld P.M., Beynon T.D., Hopewell J.W. (2011). Interaction between the biological effects of high-and low-LET radiation dose components in a mixed field exposure. Int. J. Radiat. Biol..

[14] Blue T.E., Gupta N., Wollard J.E. (1993). A calculation of the energy dependence of the RBE of neutrons. Phys. Med. Biol..

[15] Abele H., Dubbers D., Hase H., Klein M., Knöpfler A., Kreuz M., Lauer T., Märkisch B., Mund D., Nesvizhevsky V. (2006). Characterization of a ballistic supermirror neutron guide. Nucl. Instrum. Meth. A.

[16] Pedrosa-Rivera M., Ruiz-Magaña M., Porras I., Praena J., Torres-Sánchez P., Sabariego M., Köster U., Forsyth T., Soldner T., Haertlein M. (2020). Neutron radiobiology studies with a pure cold neutron beam. Nucl. Instrum. Meth. B.

[17] Auterinen I., Serén T., Anttila K., Kosunen A., Savolainen S. (2004). Measurement of free beam neutron spectra at eight BNCT facilities worldwide. Appl. Radiat. Isot..

[18] Sakurai Y., Kobayashi T. (2004). Spectrum evaluation at the filter-modified neutron irradiation field for neutron capture therapy in Kyoto University Research Reactor. Nucl. Instrum. Methods A.

[19] Narayanasamy G., Saenz D., Cruz W., Ha C.S., Papanikolaou N., Stathakis S. (2016). Commissioning an Elekta Versa HD linear accelerator. J. Appl. Clin. Med. Phys..

[20] Briesmeister J.F. (2003). X-5 Monte Carlo Team: MCNP—A General Monte Carlo N-Particle Transport Code.

[21] Bortolussi S., Postuma I., Protti N., Provenzano L., Ferrari C., Cansolino L., Dionigi P., Galasso O., Gasparini G., Altieri S. (2017). Understanding the potentiality of accelerator based-boron neutron capture therapy for osteosarcoma: Dosimetry assessment based on the reported clinical experience. Radiat. Oncol..

[22] Bedford J.L., Childs P.J., Nordmark Hansen V., Mosleh-Shirazi M.A., Verhaegen F., Warrington A.P. (2003). Commissioning and quality assurance of the Pinnacle3 radiotherapy treatment planning system for external beam photons. Br. J. Radiol..

[23] Nghia Ho. https://nghiaho.com/?page_id=1011.

[24] White D.R., Griffith R.V., Wilson I.J. (1992). Report 46. J. Int. Comm. Radiat. Units Meas..

[25] Goorley J.T., Kiger Iii W.S., Zamenhof R.G. (2002). Reference dosimetry calculations for neutron capture therapy with comparison of analytical and voxel models. Med. Phys..

[26] Fowler J.F. (1990). How worthwhile are short schedules in radiotherapy? A series of exploratory calculations. Radiat. Oncol..

[27] Bayart E., Flacco A., Delmas O., Pommarel L., Levy D., Cavallone M., Megnin-Chanet F., Deutsch E., Malka V. (2019). Fast dose fractionation using ultra-short laser accelerated proton pulses can increase cancer cell mortality, which relies on functional PARP1 protein. Sci. Rep..

